# ﻿The genus *Liocanthydrus* Guignot, 1957 (Coleoptera, Noteridae) in Argentina: new records and larval morphology

**DOI:** 10.3897/zookeys.1231.144746

**Published:** 2025-03-13

**Authors:** Juan I. Urcola, Mario E. Toledo, Stephen M. Baca, Mariano C. Michat

**Affiliations:** 1 Laboratory of Entomology, Department of Biodiversity and Experimental Biology, Faculty of Exact and Natural Sciences, University of Buenos Aires, Buenos Aires, Argentina University of Buenos Aires Buenos Aires Argentina; 2 Institute of Biodiversity and Experimental and Applied Biology (IBBEA), CONICET-University of Buenos Aires, Buenos Aires, Argentina CONICET-University of Buenos Aires Buenos Aires Argentina; 3 Department of Sustainable Crop Production (DI.PRO.VE.S.), Università Cattolica del Sacro Cuore, Piacenza, Italy Università Cattolica del Sacro Cuore Piacenza Italy; 4 Department of Entomology, LSU Agricultural Center, 404 Life Sciences Building, Baton Rouge, Louisiana 70803, USA Department of Entomology, LSU Agricultural Center Louisiana United States of America

**Keywords:** Burrowing water beetle, chaetotaxy, immatures, South America, taxonomy

## Abstract

The genus *Liocanthydrus* Guignot, 1957 is formally reported from Argentina since its first mention in an unpublished work more than 40 years ago. A single species, *L.nanops*Baca et al., 2014, is recognised, with few records in the northeast part of the country, suggesting that these may represent the southern distributional limit for the genus. The habitat, a floodplain stream in the southern Atlantic Forest, is described, where both adults and larvae were collected, as well as co-occurring taxa. The first- and third-instar larvae of *L.nanops* are described for the first time. The first instar, which was unknown for the genus, gives the chance to document its primary chaetotaxy in detail. This species presents two posterodorsal projections on the abdominal segment VIII, which represent elongations of the membranous posterolateral areas of segment VIII, where setae AB8 and AB14 are usually inserted; these two setae accompany the elongation of these regions and are therefore inserted on the projections. The projections are shared with the previously described larvae of *L.clayae* (J. Balfour-Browne, 1969) but not with those of other noterid genera, and, thus, supports this unique feature as diagnostic of *Liocanthydrus*.

## ﻿Introduction

The burrowing water beetle genus *Liocanthydrus* Guignot, 1957 currently comprises 11 medium-sized species (adult length 2.7–3.3 mm) widely distributed across the Neotropical Region, with records from Venezuela, Guyana, Suriname, French Guiana, Brazil, and Paraguay (Baca et al. 2014; Guimarães and Ferreira-Jr 2015; García et al. 2018). Unlike other noterid genera, *Liocanthydrus* occurs mainly in lotic habitats or those associated with moving water, such as pools near waterfalls (Baca et al. 2014). Morphologically, adults of this genus are characterized by the following combination of characters: (1) prosternal process very broad and truncate with a slight posteromedial projection, (2) anteroapical angle of metafemur with close, linear series of long setae, (3) pronotal margins smooth and pronotal bead broad, (4) posterior metatibial spur smooth, not serrate, and (5) female genitalia bearing short laterotergites extending posteriorly beyond bases of gonocoxae (Baca et al. 2014). Recently, the larva of a species of *Liocanthydrus* (*L.clayae* (J. Balfour-Browne, 1969)) was described for the first time (Urcola et al. 2021), and it can be easily distinguished from other known noterid genera by the presence of two posterodorsal projections on the last abdominal segment, a unique and conspicuous characteristic that facilitates differentiation from other noterids from which the larvae are known: *Canthydrus* Sharp, 1882, *Hydrocanthus* Say, 1823, *Neohydrocoptus* Satô, 1972, *Noterus* Clairville, 1806, *Phreatodytes* Uéno, 1957, *Suphis* Aubé, 1836, *Suphisellus* Crotch, 1872, *Sternocanthus* (Guignot, 1948), and *Synchortus* Sharp, 1880 (e.g. Uéno 1957; Bertrand 1972; Watts 2002; Dettner 2016; Urcola et al. 2019, 2020; Urcola and Michat 2023). However, as the first instar was not available, primary chaetotaxy could not be described and remains unknown for the genus.

In Argentina, seven noterid genera have been reported: *Hydrocanthus*, *Prionohydrus* Gómez & Miller, 2013, *Mesonoterus* Sharp, 1882, *Notomicrus* Sharp, 1882, *Suphis*, *Suphisellus*, and *Liocanthydrus* (Urcola et al. 2024). The latter genus, however, was only mentioned in a doctoral thesis (Grosso 1979), where adult morphology of a single species was documented. In that study, Grosso reported the collection of 21 specimens in Formosa Province, and identified them as *Canthydrusoctoguttatus* Zimmermann, 1921 (*Liocanthydrus*, by that time considered a subgenus within *Canthydrus*, was elevated to genus rank by Baca et al. 2014). Here, we report the presence of *L.nanops*Baca et al., 2014 in the country, and describe the larvae including primary chaetotaxy for the first time for the genus.

## ﻿Material and methods

### ﻿Material examined

One instar I and one instar III larvae of *L.nanops* were collected in association with adults at Iguazú National Park (Misiones Province, Argentina) in January 2024. The association of adults with juveniles is firmly established as no other *Liocanthydrus* species was present in the stream. *Suphisellus* and *Hydrocanthus*, the only other noterid genera found in that site, have the larvae described and can be easily ruled out by several characters (Urcola et al. 2020; Urcola and Michat 2023). Adults and larvae were collected from a stream floodplain using a dip net, preserved in 96% ethanol, and taken to the Laboratory of Entomology, Buenos Aires, Argentina (**LEBA**) for study. Additional adults from a previous collecting event in the same area (c. 1997) were found housed in LEBA and are here identified as *L.nanops*. All collected and examined material is deposited in LEBA.

### ﻿Adult morphology

As the presence of *L.octoguttatus* in Argentina is put into doubt in this paper (see below), and considering that this was the only known record of the genus in the country, we believe it prudent to provide some measurements and illustrate the habitus and male genitalia of the specimens we collected.

Male genitalia were dissected, cleared in lactic acid, mounted temporarily on slides with gel alcohol for observation, and then stored together with the specimens. Terminology of male genitalia follows Miller and Nilsson (2003) and Toledo and Negri (2024).

Habitus photographs were taken using a Nikon D800e digital camera equipped with Nikon AFS VR Micro-NIKKOR 105 mm f/2.8G IF-ED and Raynox MSN-202 lenses. Photographs of male genitalia were generated with a Leica MZ6 stereomicroscope (with Leica DMC2900 camera attached) or with an Olympus CX41 microscope (with Olympus LC30 camera attached). Images were processed using Helicon Focus 6.7.1 Pro. Drawings of male genitalia were made by tracing over photographs using the image editing software Adobe Illustrator (CC 2019).

Measurements were taken using a Leica MZ6 stereomicroscope equipped with an ocular micrometer: total length (TL), greatest width (GW), greatest width of head (HW), distance between eyes (EW), anterior pronotal width, across anterolateral angles (PNWant), posterior pronotal width, across posterolateral angles (PNWpost), total length of prosternum plus noterid platform at midline (TLVP; prosternum, metaventrite, metacoxae). The ratios TL/GW, HW/EW and PNWpost/PNWant were also calculated.

### ﻿Larval morphology

Larvae were cleared in lactic acid, dissected, and mounted on glass slides with polyvinyl-lacto-glycerol. Observations (at magnifications up to 1000×) and drawings were made using an Olympus CX41 compound microscope equipped with a camera lucida. Drawings were scanned and digitally edited using Adobe Illustrator. The methods and terms used herein largely follow those employed in a previous study of the larval morphology and chaetotaxy of the genus *Suphis* (Urcola et al. 2019). The reader is referred to that work for a complete list and additional explanations of the terms used in the present study.

## ﻿Results

### ﻿ Liocanthydrus nanops

Taxon classificationAnimaliaColeopteraNoteridae

Baca et al., 2014

68B12CA5-5154-5933-9D80-CC295A1C0FE0

Figs 1–8 , 9–12 , 16 , 18–20 , 21–27 , 28–29 , 30–33 , 34–37 , Tables 1 , 2

#### Material examined.

Argentina – Misiones Province: 2 ♂ and 4 ♀, Iguazú National Park, 25°40'S, 54°27'W, 27.IX.1997, López Ruf leg. (LEBA) • 1 larva of instar III and 19 adults, Iguazú National Park, Daniel “Pupi” Somay bird observatory, 25°42'54"S, 54°26'54"W, alt. 197 m a.s.l., 9.I.2024, Urcola leg. (LEBA) • 1 larva of instar I and 18 adults, same data except 10.I.2024 (LEBA).

#### Measurements.

TL = 2.8–3.10 mm, mean = 2.95 mm; GW = 1.35–1.50 mm, mean = 1.4 mm; TL/GW = 2.0–2.2 mm, mean = 2.1 mm; HW = 0.85–0.95 mm, mean = 0.9 mm; EW = 0.5–0.65 mm, mean = 0.55 mm; HW/EW = 1.5–1.7 mm, mean = 1.55 mm; PNWant = 0.85–1.0 mm, mean = 0.95 mm; PNWpost = 1.3–1.5 mm, mean = 1.35 mm; PNWpost/PNWant = 1.4–1.5 mm, mean = 1.45 mm; TLVP = 1.15–1.3 mm, mean = 1.2 mm.

#### Variation in adult morphology.

**Figures 1–8.**
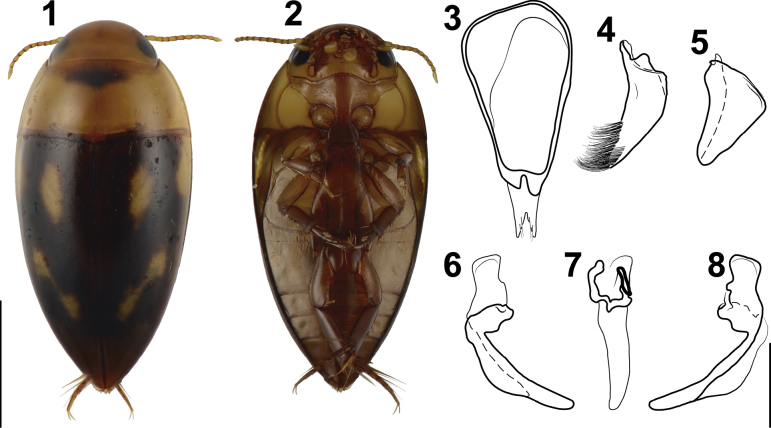
Habitus (**1, 2**) and genitalia (**3–8**) of *Liocanthydrusnanops*Baca et al., 2014, male from Argentina, Misiones Province (LEBA) **1** dorsal aspect **2** ventral aspect **3** segment IX, right lateral aspect **4** right lateral lobe, right lateral aspect **5** left lateral lobe, right lateral aspect **6** median lobe, left lateral aspect **7** median lobe, dorsal aspect **8** median lobe, right lateral aspect. Scale bars: 1.00 mm (**1, 2**); 0.25 mm (**3–8**).

Similar to what was reported by Baca et al. (2014), the specimens examined here show considerable variation in color. Many of the specimens exhibit a yellow head and pronotum, whereas in others these parts are reddish brown. The dark macula on the anteromedial region of the pronotum (Fig. 1) is absent in some individuals. Most of the specimens have dark elytra with clearly visible yellow spots (Fig. 1), while in others the elytra are dark reddish brown and the spots are only slightly lighter. The specimens collected in 1997 have a reddish-brown head and pronotum, the latter lacking the dark anteromedial macula, and the elytra are slightly darker with the spots barely visible (similar in color to the head and pronotum). However, examination of the aedeagus revealed the same diagnostic characters as reported by Baca et al. (2014) and the other specimens collected here. Finally, the two males collected in 1997 have the anterodorsal margin of sternite IX widened, similar to that reported by Grosso (1979) for specimens identified as *L.octoguttatus*. However, we observed that this character is variable in the rest of the material examined, with this widening absent in most specimens (Fig. 3).

**Figures 9–12.**
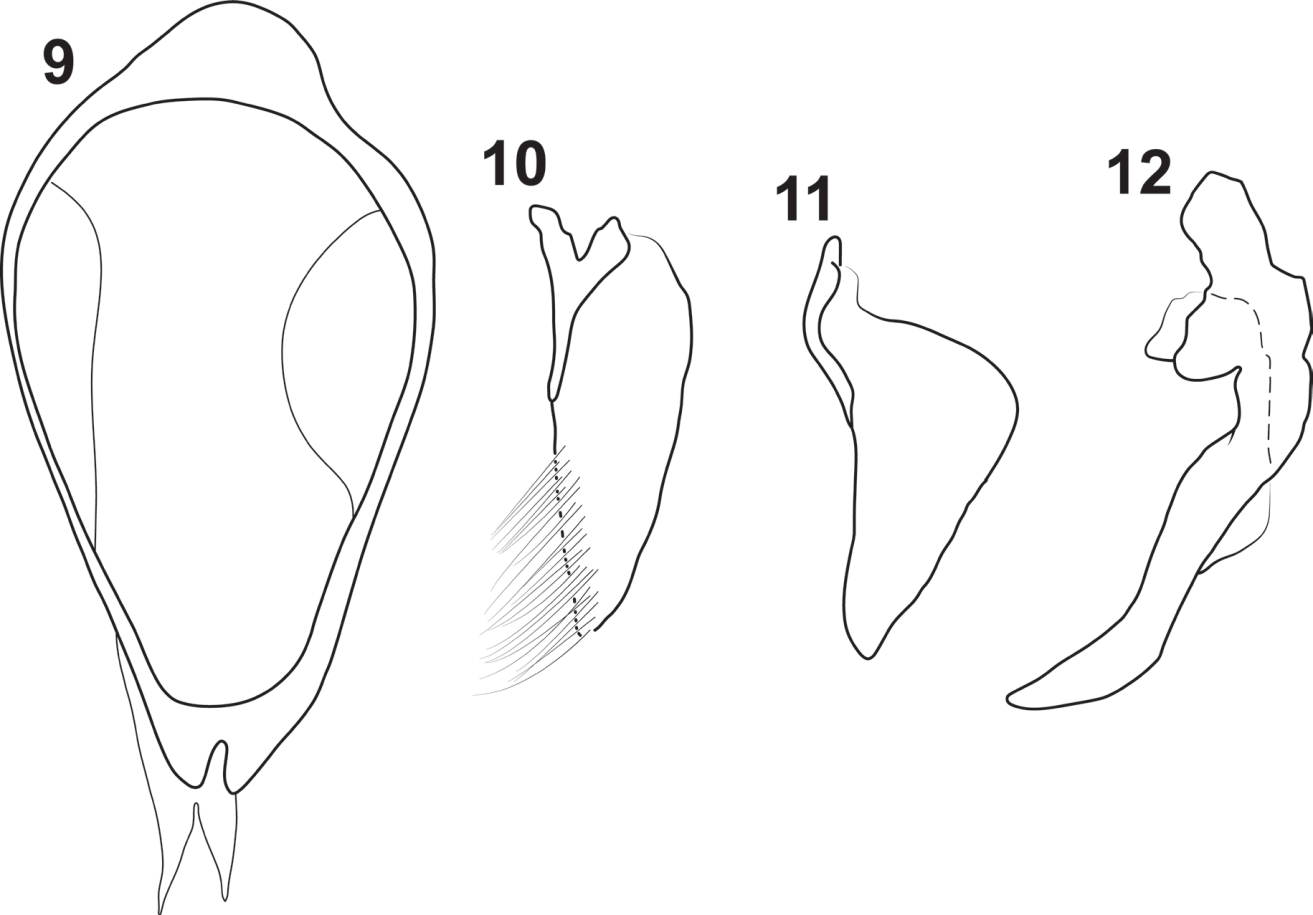
Male genitalia of *Liocanthydrusnanops*Baca et al., 2014, modified from drawings in Grosso (1979)**9** segment IX, right lateral aspect **10** right lateral lobe, right lateral aspect **11** left lateral lobe, right lateral aspect **12** median lobe, right lateral aspect. Scale bar: 0.5 mm.

**Figures 13–16.**
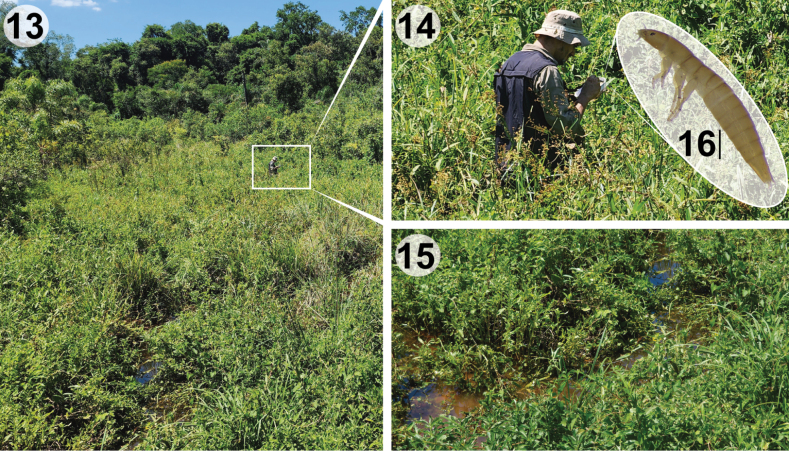
Habitat of *Liocanthydrusnanops*Baca et al., 2014, Iguazú NP, Misiones Province, Argentina **13** general view of the stream’s flooding site **14** collecting of beetles **15** details of the stream **16** instar III larva of *L.nanops*. Scale bar: 0.5 mm.

#### Remarks.

**Figure 17.**
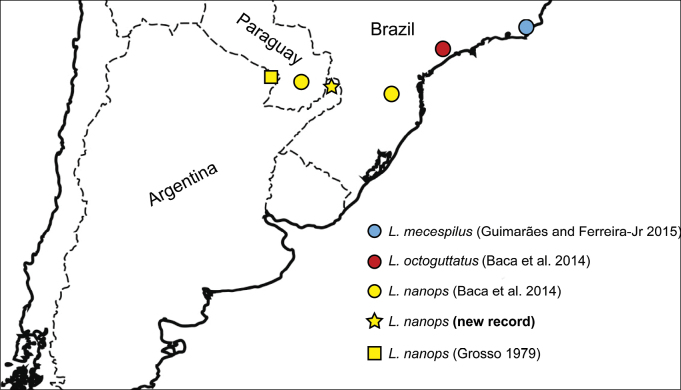
Known distributional data for the southernmost *Liocanthydrus* species with references of records.

Based on Grosso’s (1979) redescription and drawings of the male genitalia of the specimens alleged to be *L.octoguttatus* (Figs 9–12, modified from original drawings), we can observe that the left lateral lobe has a well-projected distal angle (Fig. 11). The only known species of *Liocanthydrus* in which the left lateral lobe has this shape is *L nanops* (Baca et al. 2014) (Fig. 5), which is also the only species distributed near the area where the specimens studied by Grosso were collected (Fig. 17). This evidence, summed to the shape of the apex of the median lobe in lateral view (Fig. 12) led us to conclude that the specimens studied by Grosso belong to *L.nanops*. It should be noted that, when Grosso identified his material, *L.nanops* had not yet been described. Grosso likely relied on Zimmermann’s (1921) treatment of *L.octoguttatus*, which lacks a description of the male genitalia (a crucial feature to recognize that it belongs to a distinct species given the very similar external appearance of both species).

**Figures 18–20.**
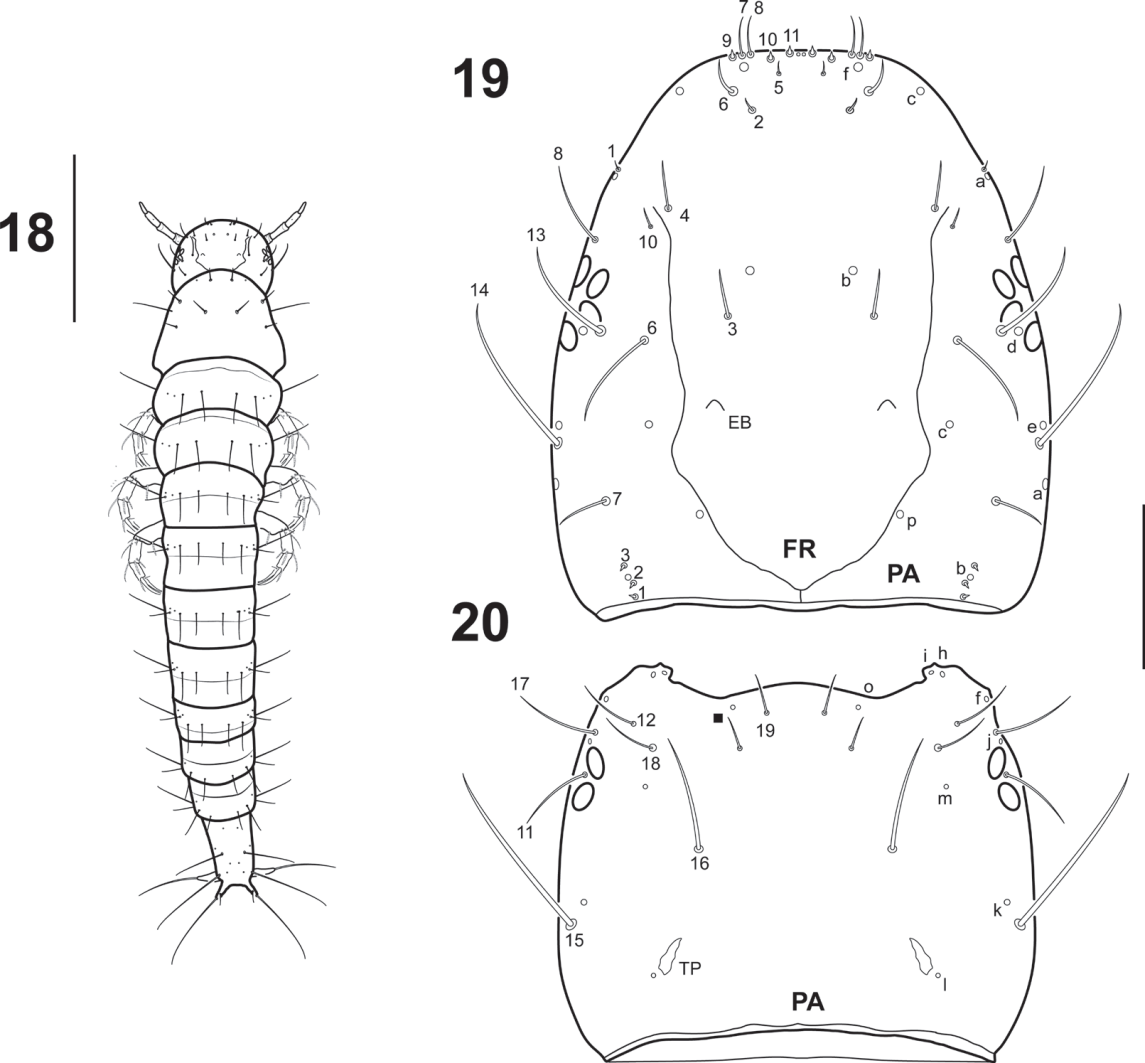
*Liocanthydrusnanops*Baca et al., 2014, instar I **18** habitus, dorsal aspect **19** cephalic capsule, dorsal aspect **20** cephalic capsule, ventral aspect. Numbers and lowercase letters indicate primary setae and pores, respectively. Solid square indicates additional seta. EB: egg burster, FR: frontoclypeus, PA: parietal, TP: tentorial pit. Scale bars: 0.50 mm (**18**); 0.10 mm (**19, 20**).

**Figures 21–27.**
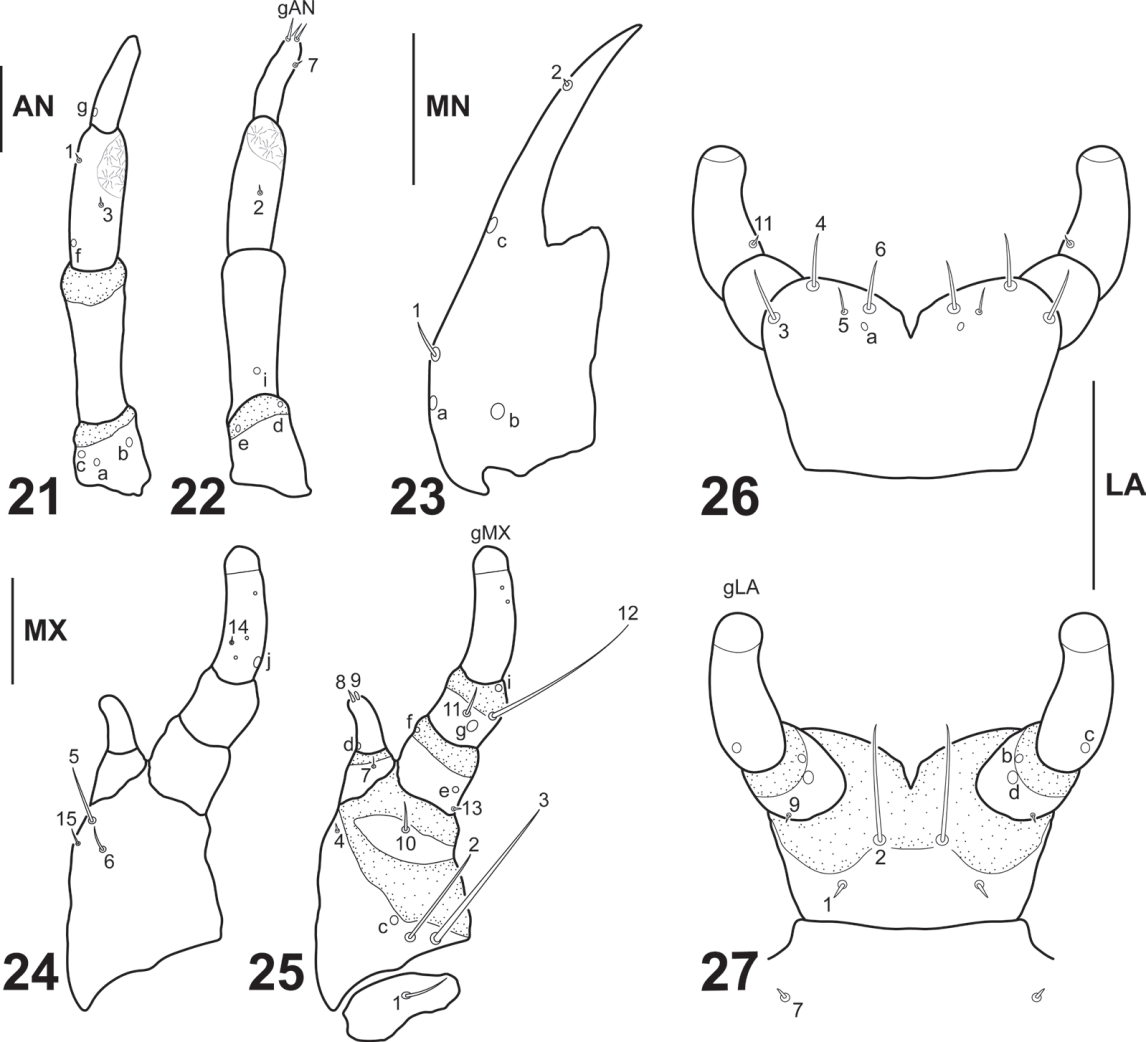
*Liocanthydrusnanops*Baca et al., 2014, instar I **21** left antenna, dorsal aspect **22** right antenna, ventral aspect **23** left mandible, dorsal aspect **24** right maxilla, dorsal aspect **25** left maxilla, ventral aspect **26** labium, dorsal aspect **27** labium, ventral aspect. Numbers and lowercase letters indicate primary setae and pores, respectively. AN: antenna, LA: labium, MN: mandible, MX: maxilla. Scale bars: 0.04 mm.

**Figures 28–29.**
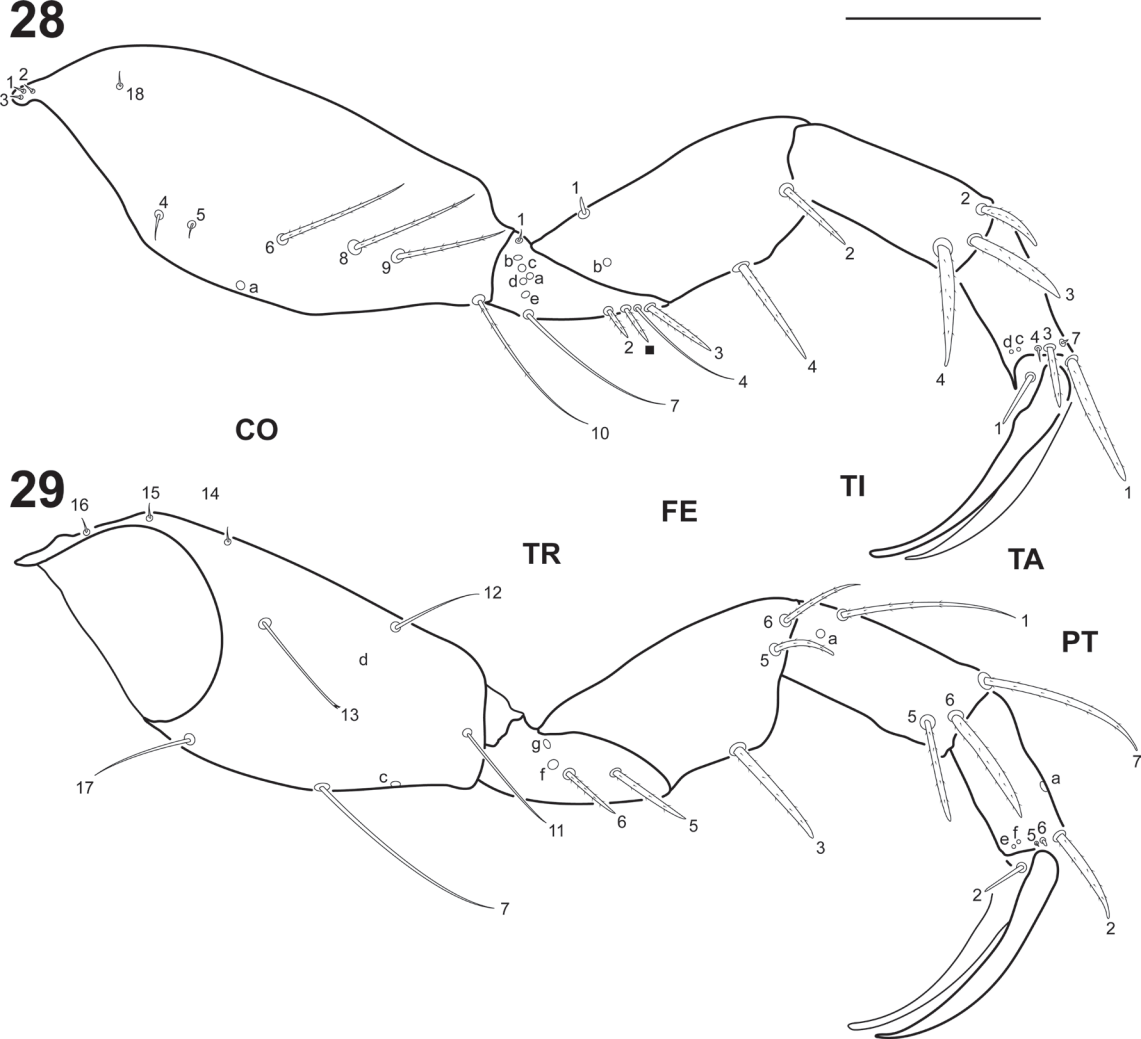
*Liocanthydrusnanops*Baca et al., 2014, instar I **28** left metathoracic leg, anterior aspect **29** right metathoracic leg, posterior aspect. Numbers and lowercase letters indicate primary setae and pores respectively. Solid square indicates additional seta. CO: coxa, FE: femur, PT: pretarsus, TA: tarsus, TI: tibia, TR: trochanter. Scale bar: 0.10 mm.

#### Habitat and co-occurring taxa.

Adults and larvae of *L.nanops* were collected in a stream floodplain with other noterid species: *Hydrocanthussocius* Sahlberg, 1844, *Suphisellusbalzani* (Régimbart, 1889), and *S.rufipes* (Sharp, 1882). The sampling site was mostly exposed to sunlight, had a muddy bottom, shallow depth, slow current, cool water, and abundant emergent vegetation (Figs 13–15).

#### Distribution.

Argentina (Formosa, Misiones) (new record), Brazil, and Paraguay (Fig. 17).

#### Description of larva.

**Instar I** (Figs 18–33).

**Figures 30–33.**
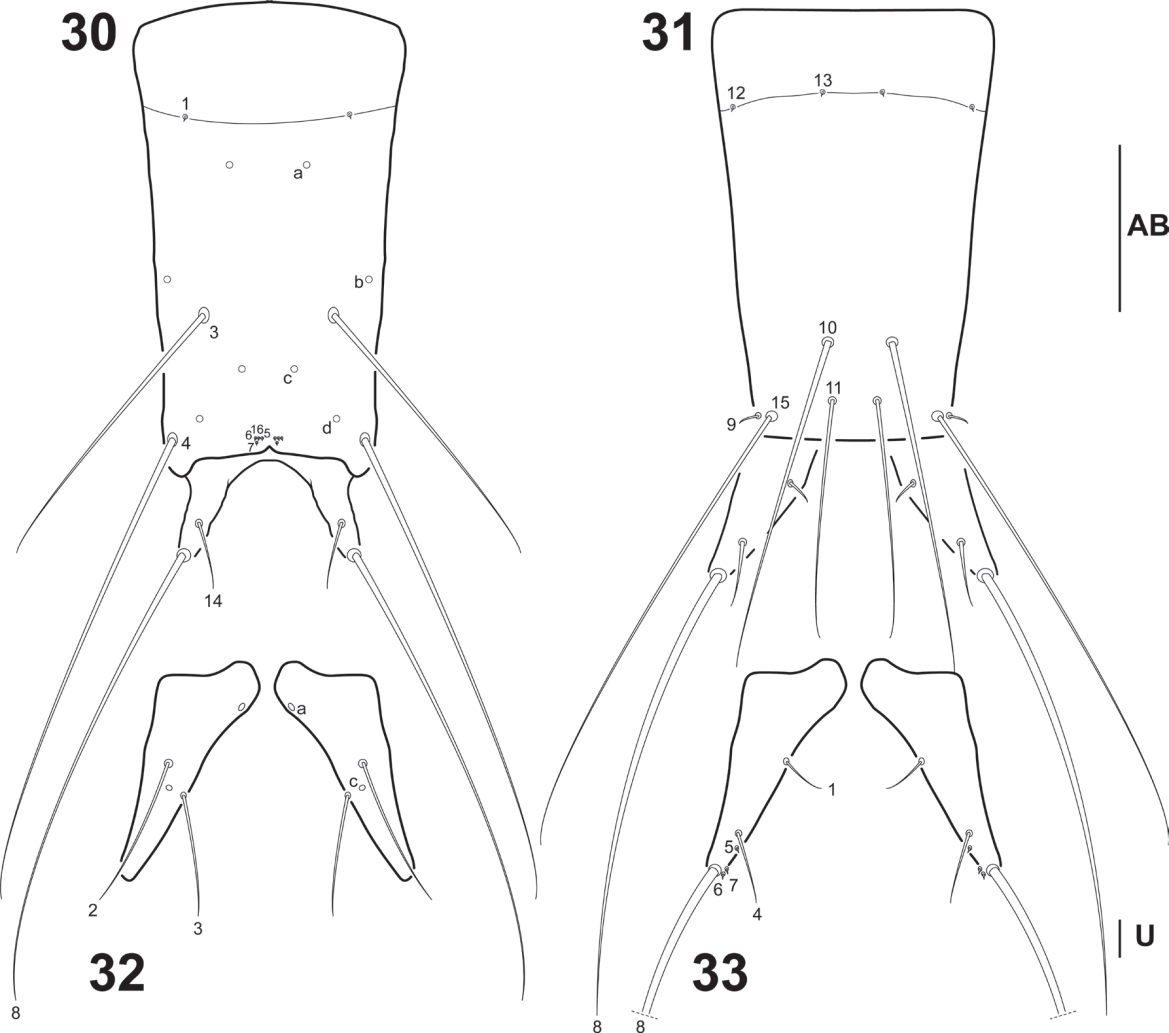
*Liocanthydrusnanops*Baca et al., 2014, instar I **30** abdominal segment VIII, dorsal aspect **31** abdominal segment VIII and urogomphi, ventral aspect **32** urogomphi, dorsal aspect **33** urogomphi, ventral aspect. Numbers and lowercase letters indicate primary setae and pores, respectively. AB: abdominal segment VIII, UR: urogomphus. Scale bars: 0.10 mm (**30, 31**); 0.02 mm (**32, 33**).

***Color*.** Entirely testaceous.

***Body*.** Elongate, nearly parallel sided (Fig. 18). Measurements and ratios that characterize the body shape are given in Table 1.

**Table 1.** Measurements and ratios for the larval instars of *Liocanthydrusnanops*Baca et al. 2014 (*n* = 1).

Measure	Instar I	Instar III	Measure	Instar I	Instar III
TL (mm)	1.97	3.51	MP1/MP3	0.50	0.69
MW (mm)	0.38	0.63	MP2/MP3	0.60	0.62
HL (mm)	0.34	0.56	MP/LP	2.10	1.20
HW (mm)	0.31	0.56	LP1/LP2	0.67	0.67
FRL (mm)	0.34	0.54	L3 (mm)	0.74	1.27
OCW (mm)	0.25	0.51	L3/L1	1.25	1.27
HL/HW	1.10	1.00	L3/L2	1.09	1.10
HW/OCW	1.24	1.08	L3/HW	2.37	2.31
COL/HL	0.02	0.02	CO/FE (L3)	1.85	1.70
FRL/HL	0.99	0.98	TI/FE (L3)	0.72	0.63
A/HW	0.60	0.53	TA/FE (L3)	0.57	0.44
A1/A3	0.63	0.59	CL/TA (L3)	1.38	1.44
A2/A3	1.05	1.29	LAS (mm)	0.27	0.70
A4/A3	0.73	0.53	LAS/HW	0.87	1.26
MNL/MNW	3.25	2.53	U (mm)	0.13	0.17
MNL/HL	0.38	0.35	U/LAS	0.48	0.25
A/MP	1.79	1.93	U/HW	0.42	0.31
GA/MP1	1.20	1.00			

***Head*.** Prognathous; cephalic capsule (Figs 19, 20) slightly longer than broad; maximum width posterior to stemmata; slightly narrowed posteriorly; occipital foramen large; coronal suture very short; ecdysial suture U-shaped; tentorial pits visible postero-ventrally, well separated from each other and from occipital foramen; six lateral stemmata arranged in two curved vertical rows at each side. Frontoclypeus elongate, roughly subovate, anterior margin rounded, with two spine-like egg bursters on basal third. ***Antenna*** (Figs 21, 22). Short, robust, shorter than maximum head width, composed of four antennomeres; A2 and A3 longest, subequal; A3 with a rugged area on distal portion; A4 approximately 3/4 length of A3; A1 shortest. ***Mandible*** (Fig. 23). Symmetrical, short, basal half broad, inner margin with strong subrectangular process, distal half slender, curved inwards, narrowing to pointed apex, inner margin smooth. ***Maxilla*** (Figs 24, 25). Cardo small, suboval; stipes well developed, subtrapezoidal, bearing a galea on distal inner margin and a palpus on distal outer margin; galea well developed, composed of two galeomeres, GA2 more slender and subequal in length to GA1; palpifer not cleary differentiated from stipes, more evident in ventral view; palpus short, robust, composed of three palpomeres, MP1 shortest, MP3 longest. ***Labium*** (Figs 26, 27). Prementum well developed, subrectangular, somewhat broader than long, anterior margin narrowly indented medially; palpus short, robust, composed of two palpomeres, LP2 longer than LP1.

**Figures 34–37.**
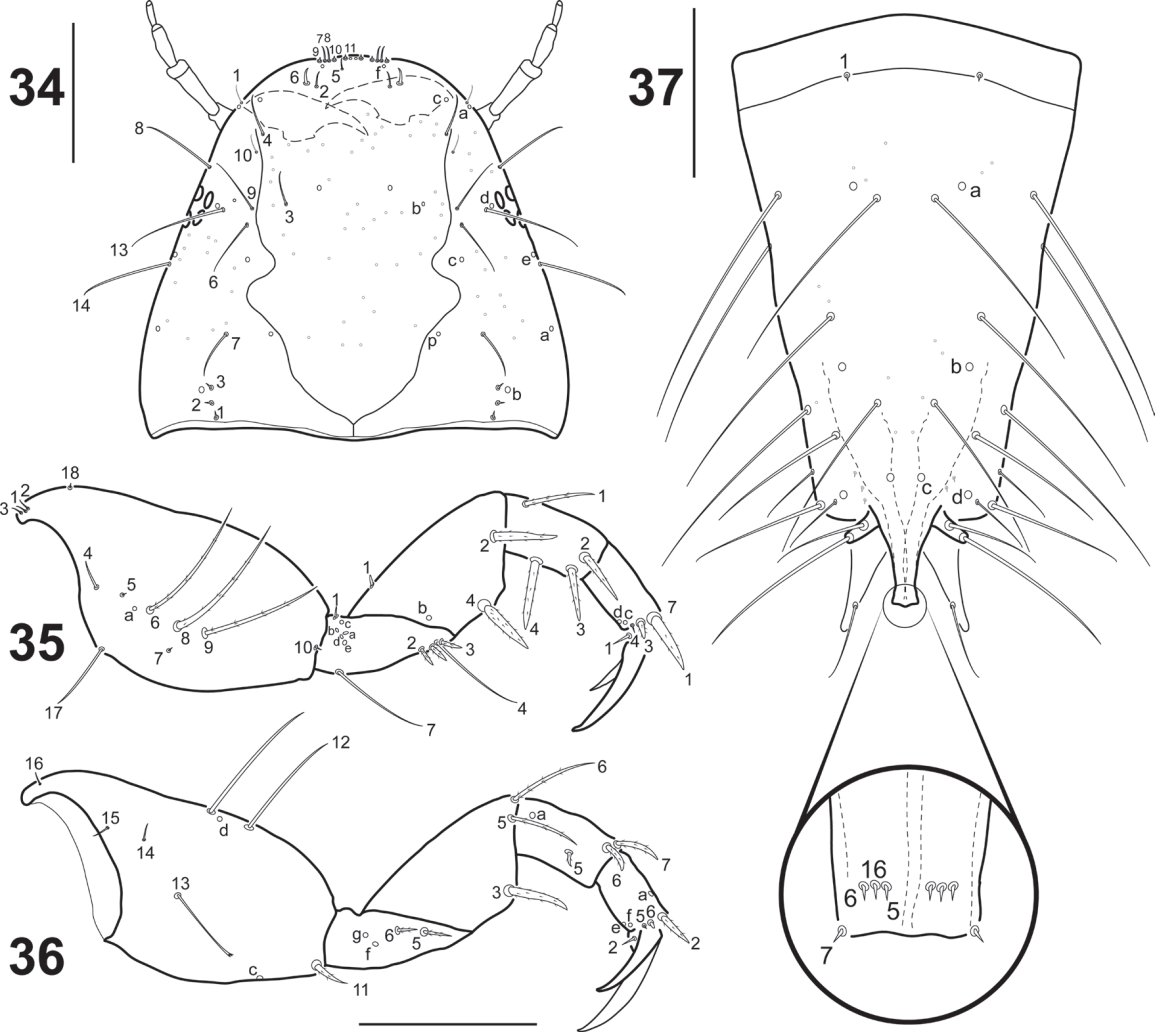
*Liocanthydrusnanops*Baca et al., 2014, instar III **34** head, dorsal aspect **35** left prothoracic leg, anterior aspect **36** right prothoracic leg, posterior aspect **37** abdominal segment VIII, dorsal aspect. Numbers and lowercase letters indicate primary setae and pores, respectively (some setae on abdominal segment VIII could not be identified). Scale bars: 0.20 mm.

***Thorax*.** Terga fully sclerotised, convex (Fig. 18); pronotum about as long as meso- and metanotum combined, meso- and metanotum subequal in length, approximately as wide as pronotum; protergite subrectangular, lateral margins rounded, more developed than meso- and metatergite; meso- and metatergite with anterotransverse carina; ecdysial line absent. ***Legs*** (Figs 28, 29). Short, robust, composed of six articles, L1 shortest, L3 longest; coxa broad, elongate, trochanter lacking annulus, femur, tibia and tarsus short, subcylindrical, pretarsus with two long, slender, slightly curved claws, posterior claw slightly shorter than anterior claw.

***Abdomen*.** Eight-segmented (Fig. 18); segments I–VIII completely sclerotised, ring-like, progressively narrowing to apex, with anterotransverse carina; segment VIII (Figs 30, 31) the longest and narrowest, with two terminal dorsal projections, lacking a U-shaped wavy membranous area ventrally, contiguous to urogomphi; siphon absent. ***Urogomphi*** (Figs 32, 33). Short, cylindrical, visible in dorsal view (Fig. 18), not fused to each other.

***Chaetotaxy*.** Similar to that of *L.clayae* (see Urcola et al. 2021) except for the following features: seta FR1 very short (Fig. 19); seta PA9 absent (Fig. 19); seta AN1 inserted more distally (Fig. 21); seta MN1 short (Fig. 23); MP3 with several minute sensilla on surface (Figs 24, 25); pore URa located on dorsal surface (Fig. 32). Setae on abdominal segment VIII were not named in instar II of *L.clayae* due to the presence of secondary setae. Therefore, they are detailed here for *L.nanops*: dorsal surface of segment VIII with one seta (AB1) on basal region, one seta (AB3) on distal third and one long (AB4) and four minute (AB5, AB6, AB7, AB16) setae apically (Fig. 30); each terminal dorsal projection with one short medial seta (AB14) and one long apical seta (AB8); ventral surface of segment VIII with two setae (AB12, AB13) on basal region and four setae (AB9, AB10, AB11, B15) on distal region (Fig. 31).

**Instar III** (Figs 16, 34–37)

As for instar I except for the following features:

***Body*.** Measurements and ratios that characterise body shape are shown in Table 1.

***Head*.** Egg bursters absent; A2 longer than A3; A4 shortest, approximately 1/2 length of A3; mandible more robust, process less prominent (Fig. 34).

***Abdomen*.** Siphon relatively long, slender, apex truncated (Fig. 37).

***Chaetotaxy*.** Frontoclypeus with 14–21 minute secondary setae on anterior half and 3–4 minute secondary setae on posterior half; dorsal surface of parietal with seta PA9 present (inserted close to seta PA6), 0–4 minute secondary setae on anterior portion and 10–13 minute secondary setae on posterior portion (Fig. 34); ventral surface of parietal with 10–13 minute secondary setae on anterior half and 1–2 minute secondary setae on posterior half; secondary leg setation detailed in Table 2 and Figs 35, 36; abdominal segments I–VII with several secondary setae; dorsal surface of abdominal segment VIII (Fig. 37) with three elongate hair-like secondary setae and 2–3 minute secondary setae on anterior portion, 2–3 minute secondary setae on medial portion, and five hair like-like secondary seta and 4–5 minute secondary setae on posterior portion; ventral surface of abdominal segment VIII with two elongate hair-like secondary setae on anterior portion, one elongate hair-like secondary seta on medial portion, and one short and one long secondary setae on posterior portion.

**Table 2.** Number and position of secondary setae on the legs of larvae of *Liocanthydrusnanops*Baca et al. 2014. Numbers between slash marks refer to pro-, meso-, and metathoracic leg, respectively. A = anterior, PD = posterodorsal, PV = posteroventral; Total = total number of secondary setae on the article (i.e., excluding primary setae) (*n* = 1).

Article	Position	Instar III
Coxa	A	0 / 2 / 2
	PD	1 / 1 / 1
	PV	0 / 1 / 1
	Total	1 / 4 / 4

#### Remarks.

When comparing the first and third instars of *L.nanops* with the supposedly third instar of *L.clayae* (Urcola et al. 2021), we can conclude that the larva of this latter species is actually a second instar. This conclusion is based on the following evidence: the head of the larva of *L.clayae* (as expressed in the head width) exhibits an intermediate size between the first and third instars of *L.nanops*; the mandibles in *L.clayae* are not as robust as those of the third instar of *L.nanops*; and the siphon in *L.clayae* is more developed than that of the first instar of *L.nanops* but not as strongly developed as that of the third instar of this species. Regarding chaetotaxy, seta PA9 is absent on the parietal of the first instar of *L.nanops*. This conspicuous sensillum, however, is present in the third instar of this species (Fig. 34), as well as in all noterid larvae known in detail (e.g. Urcola et al. 2019, 2020, 2021). Since we examined only a single specimen of each instar of *L.nanops*, we prefer not to consider the absence of PA9 in instar I as a diagnostic character for the species until more material can be studied.

## ﻿Discussion

In this study we document the finding of *L.nanops* in Argentina and thus formally report the presence of the genus *Liocanthydrus* in the country after its first mention in an unpublished work more than 40 years ago (Grosso 1979). We also postulate that the only series of specimens previously known from the country, identified as *L.octoguttatus* (Grosso 1979), is conspecific with *L.nanops*, thus excluding *L.octoguttatus* from the Argentine fauna. These findings significantly enhance our understanding of the distribution of this genus in the southern limit of its range. Additional samplings, however, may reveal the presence of this genus in other areas of northeastern Argentina and southern Brazil and Paraguay.

The larvae of *L.nanops* are described here for the first time, raising to two the number of species of *Liocanthydrus* with larvae known in detail. Even though only a single specimen of each instar I and III was examined, we compared both the morphometric and chaetotaxic features with those of the second instar of *L.clayae* (misidentified as third instar in Urcola et al. 2021). The larvae of both species share the following characteristics: (1) an elongated body, (2) the posterior tentorial pits not contiguous with the occipital foramen, (3) the seta AN7 inserted distally on antennomere 4, (4) the inner dorsal margin of the mandible not serrate, (5) the absence of a U-shaped wavy membranous area ventrally on abdominal segment VIII, (6) the presence of two posterodorsal projections on the abdominal segment VIII, and (7) the urogomphi not fused along inner margin. The posterodorsal projections of the abdominal segment VIII, first described in Urcola et al. (2021), are so far unique within Noteridae and represent posterior elongations of the membranous posterolateral areas of segment VIII, where the setae AB8 and AB14 are usually inserted (see for example their insertion in *Suphis* and *Hydrocanthus*, Urcola et al. 2019, 2020). Consequently, setae AB8 and AB14 accompany the elongation of these regions and are therefore part of the posterior projections that characterize *Liocanthydrus* larvae.

## Supplementary Material

XML Treatment for Liocanthydrus nanops

## ﻿Additional information

### Author ORCIDs

Juan I. Urcola https://orcid.org/0000-0002-5684-2464

Mario E. Toledo https://orcid.org/0000-0001-9295-3711

Stephen M. Baca https://orcid.org/0000-0002-0957-302X

Mariano C. Michat https://orcid.org/0000-0002-1962-7976

### Data availability

All of the data that support the findings of this study are available in the main text.
